# Traditions in Spider Monkeys Are Biased towards the Social Domain

**DOI:** 10.1371/journal.pone.0016863

**Published:** 2011-02-23

**Authors:** Claire J. Santorelli, Colleen M. Schaffner, Christina J. Campbell, Hugh Notman, Mary S. Pavelka, Jennifer A. Weghorst, Filippo Aureli

**Affiliations:** 1 Department of Psychology, University of Chester, Chester, United Kingdom; 2 Department of Anthropology, California State University, Northridge, California, United States of America; 3 Anthropology, Centre for Work and Community Studies, Athabasca University, Athabasca, Alberta, Canada; 4 Department of Anthropology, University of Calgary, Calgary, Alberta, Canada; 5 Natural History Museum and Biodiversity Research Center, University of Kansas, Lawrence, Kansas, United States of America; 6 Research Centre in Evolutionary Anthropology and Palaeoecology, School of Natural Sciences and Psychology, Liverpool John Moores University, Liverpool, United Kingdom; University of Jyväskylä, Finland

## Abstract

Cross-site comparison studies of behavioral variation can provide evidence for traditions in wild species once ecological and genetic factors are excluded as causes for cross-site differences. These studies ensure behavior variants are considered within the context of a species' ecology and evolutionary adaptations. We examined wide-scale geographic variation in the behavior of spider monkeys (*Ateles geoffroyi*) across five long-term field sites in Central America using a well established ethnographic cross-site survey method. Spider monkeys possess a relatively rare social system with a high degree of fission-fusion dynamics, also typical of chimpanzees (*Pan troglodytes*) and humans (*Homo sapiens*). From the initial 62 behaviors surveyed 65% failed to meet the necessary criteria for traditions. The remaining 22 behaviors showed cross-site variation in occurrence ranging from absent through to customary, representing to our knowledge, the first documented cases of traditions in this taxon and only the second case of multiple traditions in a New World monkey species. Of the 22 behavioral variants recorded across all sites, on average 57% occurred in the social domain, 19% in food-related domains and 24% in other domains. This social bias contrasts with the food-related bias reported in great ape cross-site comparison studies and has implications for the evolution of human culture. No pattern of geographical radiation was found in relation to distance across sites. Our findings promote *A. geoffroyi* as a model species to investigate traditions with field and captive based experiments and emphasize the importance of the social domain for the study of animal traditions.

## Introduction

Traditions in wild populations are defined as “enduring behavior patterns shared among members of a group that depend to a measurable degree on social contributions to individual learning, resulting in shared practices among members of a group” [1] (p. 3). Evidence for traditions is often initially achieved through the documentation of between-group behavioral variation, once ecological and genetic explanations are excluded [2]–[9]. A crucial aspect of a tradition is that it derives from socially learned information (i.e. the ability to extract information from observing, or interacting with, another individual or its products [10]), and that it is not genetically inherited or individually learned information [11], [12]. Under the appropriate circumstances, adaptations as a result of social learning can be more rapid than those resulting from natural selection and less risky than those obtained through individual trial and error learning [13], [14]. Although criticized for being unable to definitively rule out the influence of genes or ecology [15]–[17], cross-site studies have the advantage of ensuring that behaviors are considered within the context of a species' ecology and evolutionary adaptations [2].

Spider monkeys (*Ateles* spp.) are well suited for a study of traditions because they possess several characteristics thought to promote social learning. Firstly, infants and juveniles are slow to develop compared to monkeys of a similar size and lifespan [18], providing prolonged exposure to maternal skills. Secondly, spider monkeys are socially tolerant [19], a feature predicted to facilitate social learning [20], [21]. Thirdly, spider monkeys live in communities characterized by a high degree of fission-fusion dynamics, in which individuals split and merge into subgroups of variable composition [19]. This social system is relatively rare among mammals, but it is shared with chimpanzees (*Pan troglodytes*), bonobos (*P. paniscus*) and humans (*Homo sapiens*) [22]–[24]. Milton [25], [26] pointed out that the foraging patterns of species with such fluid fission-fusion dynamics would also place them under great pressure to develop key skills, including enhanced communication systems for rapid recognition and greeting behaviors to facilitate reunions cf. [24]. The behavioral repertoire of *A. geoffroyi* comprises a number of gestures including embracing and pectoral sniffing, that likely function as greetings [19], [27], [28]. In addition, spider monkeys use a range of substrate marking behaviors for delayed olfactory communication [29]–[31], which may convey information between community members that visit the same location in separate subgroups at different times. Variation across communities in greeting and marking behaviors may occur and additionally serve to convey community identity, making them ideal potential behaviors for traditions.

With the exception of Perry et al. 's [5] study on capuchin monkeys (*Cebus capucinus*), documenting variation in behaviors involving extractive foraging and tool-use has been a main focus of previous cross-site primate studies [3], [6], [32]–[34]. There is a discrepancy between this focus and the awareness that many human traditions involve social behavior [35]. This discrepancy may be due to tool use or object manipulation being clearly identifiable [36], and object function being immediately apparent [37].

Of the three features that are fundamental for material culture (*sensu* McGrew [38]), including extractive foraging, dexterous manipulation and tolerant gregariousness [21], spider monkeys rarely show the first two. They are ripe fruit specialists [39], [40] and their nutritional needs are largely met by plant substrates easily accessed in the canopy [41]. Dexterous manipulation in spider monkeys is likely limited due to a dramatic reduction of the pollex or opposable thumb [42] and to them not having separate control of individual fingers [43], [44] (Figure S1), which would make the firm gripping of objects problematic. These two hand adaptations are thought to afford *Ateles* with the skills needed for their highly arboreal lifestyle and specialized locomotion [42], [43]. Consequently, these anatomical and dietary adaptations indicate that spider monkeys would be unlikely to engage in many behaviors relating to extractive foraging or tool use.

To our knowledge, no systematic study of traditions across different populations of spider monkeys has been carried out, although several publications suggest potential behaviors that could show inter-community variation and patterns of transmission via social learning, including meliponid bee (*Scaptotrigona* spp.) eating [45], [46], self-anointing behavior with plant substrates [30], [47], terrestrialism [48] and self-scratching using sticks [49]. These reports all document potential community variation in *Ateles* behavioral repertoire, but offer no indication that these behaviors are either learned socially or, in the case of tool-use, are being successfully transmitted between individuals. The aim of our study was to provide the first systematic evidence for traditions in spider monkeys, using a large sample of candidate behaviors across five distantly located populations of the same species with special emphasis on the domains in which the traditions occur. The similarity of their social system with that of humans [19], [23] makes the investigation of traditions in the social domain particularly relevant. First, we predicted that evidence for traditions within the social domain of spider monkeys would be more prevalent than in other domains including material traditions. Second, we predicted that candidate behaviors for traditions would likely incorporate behaviors related to community identity, such as greeting and marking behaviors.

## Results

A survey list of 62 behaviors (Table S1) was compiled and used by the authors to document the occurrence and prevalence of each behavior within each monkey community at the five long-term field sites. This method allowed for a comparison of behavioral variance across sites while minimizing ecological and genetic differences (see Methods). In keeping with the original methodology used by Whiten et al. [3], [32], and subsequently followed by Panger et al. [33] and van Schaik et al. [6], each behavior was classified into one of the following categories: customary, habitual, present, absent, ecological explanation and unknown (see Method for definitions).

### Behaviors that failed criteria for traditions

Forty of the proposed 62 behavior variants failed to meet the necessary criteria for traditions [3] for four reasons presented in bands A–D of Table S2. Ten of these behaviors were absent across all five sites (band A, Table S2). Three such behaviors were related to the consumption of non-vegetative matter and were included in the original questionnaire as other *Ateles* species consume them [39], [45]. The remaining seven behavior variants in this band were included in the survey list as they occurred in at least one site, but did not meet the ‘present’ criteria.

Six behavior variants were absent from the majority of sites, but clearly present at one or two, although not to the extent of being habitual or customary (band B, Table S2). It is possible that these behaviors are examples of current innovations at these sites; however, before social transmission can be inferred it seems reasonable that more than two individuals are required to exhibit such behaviors [33]. Four further behaviors were shown to be habitual or customary at some sites, but their absence at the other sites could be explained by ecological factors, or the existence of substrates used to perform the behavior at a site was unknown (band C, Table S2). Although social learning of these four behaviors cannot be ruled out, the currently available data are inconclusive for their inclusion as traditions. For example, one of these four behaviors, ‘raiding’, involves males walking on the ground single file in silence into the territory of neighboring communities [50]. Raiding has been observed by all males of the Eastern community multiple times at the Punta Laguna site and therefore deemed customary. It is unclear whether raiding occurs at the Corcovado site because although the subjects' actions were similarly described, no inter-community encounter was observed, and it is unknown how deep into the neighboring territory these incursions were. More importantly, as raiding likely occurs as a response to key socioecological conditions, such as reduced mating opportunities and strong male-male coalitions [50], its absence at other sites could be due to these conditions not being met, rather than an absence due to lack of social transmission. Thus, without clear evidence of how other communities respond to similar socioecological conditions, it would be premature to categorize raiding as a tradition.

Twenty behavior variants were observed across all sites (i.e., were ‘universal’ behaviors [3], [32]), although with differing degrees of prevalence among community members (band D, Table S2). These universal behaviors included a number of greetings, which are characteristic of spider monkey repertoires [19]. In addition, there were also a number of behaviors used for threats or aggressive escalation. As Whiten et al. [32] suggested from observing similar behavior patterns across chimpanzee communities, there is no way of knowing if these are genetically-based species-specific behaviors or traditions that have arisen independently at each site. They could be examples of traditions that have become homogenized within communities due to conformity and led to reduced inter-community variation [51], [52]. However, the ethnographic record cannot establish the origin of this pattern and, as a consequence of the absence of variation across sites, there was a lack of direct evidence for traditions.

### Behaviors that met criteria for traditions

**Figure 1:**
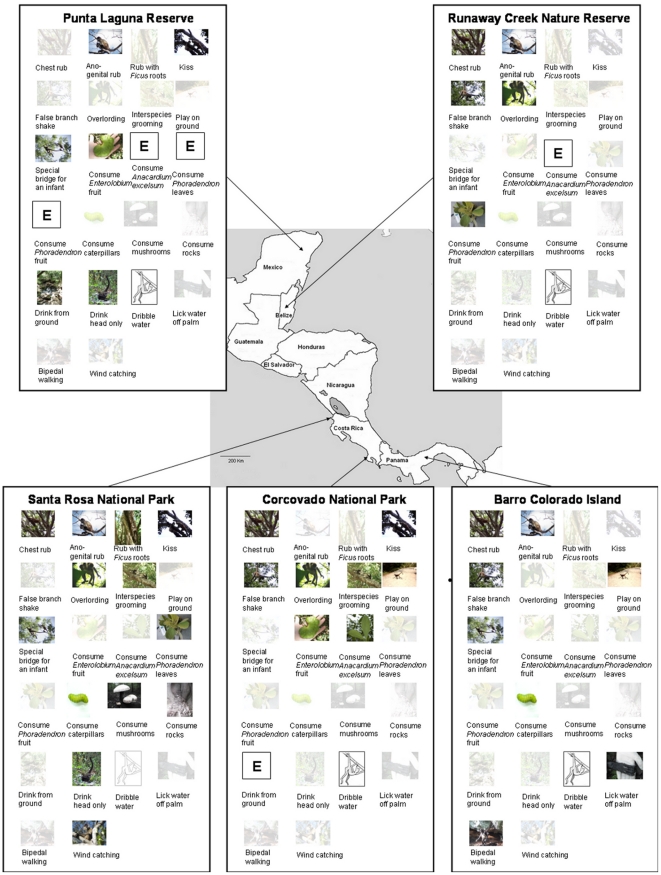
Distribution of traditions observed across the five study sites. The photograph of a behavior indicates its presence at the customary or habitual level at the field site. A faded photograph of a behavior indicates its absence or occurrence only at the present level at the field site. A missing photograph with an ‘E’ indicates the absence of the behavior at a field site due to ecological reasons (Photographs by Claire J. Santorelli and Luisa Rebecchini and drawing by Claire J. Santorelli).

The remaining 22 behaviors showed variation in their occurrence across the study sites ranging from absent through to customary with absence in at least one site not due to an ecological explanation (Table 1; Figure 1). These patterns of occurrence across sites provide evidence that these behaviors are not species-specific or absent due to ecological reasons, and they best fit the criteria for traditions. The number of traditions was slightly greater within the three Southern sites than the two Northern sites. Spider monkeys at the Northern sites of Runaway Creek and Punta Laguna showed six and seven traditions respectively, whereas individuals at the Southern sites of Barro Colorado and Corcovado showed nine each, and Santa Rosa the most with thirteen.

**Table 1:** Prevalence of the 22 behaviors which met criteria for traditions across the five sites.

Domain	Behavior†	Prevalence				
		BCI	CV	SR	RC	PL
Social	Chest rub	H	H	C	H	A
	Ano-genital rub	H	A	H	H	H
	Rub with *Ficus* root	A	A	H	P	A
	Kiss	A	H	C	P	H
	False branch shake	H	C	P	H	A
	Overlording	A	C	H	H	P
	Interspecies grooming	A	H	H	A	A
	Play on ground	H	H	A*	A	P
	Special bridging for an infant	H	P	H	A	H
Food	Consume *Enterolobium cyclocarpum* fruit	A	C	A	A	C
	Consume *Anacardium excelsum* pith	A	C	A	E	E
	Consume *Phoradendron* leaves	A	A	H	A	E
	Consume *Phoradendron* fruit	A	A	A	H	E
	Consume caterpillars/larvae	H	A	H	A	P
	Consume mushrooms	A	A	H	A	A
	Consume rocks	A	A	H	A	A
Other	Drink from ground waterhole/lake	A	E	P	A	C
	Drink using head only	+	+	H	A	C
	Dribble water into mouth	H	C	A	C	H
	Lick water off palm	H	A	A	A	A
	Bipedal walking	C	P	P	A	P
	Wind catching	A	A	H	A	A

C =  customary; H =  habitual; P =  present; A =  absent; E =  ecological explanation; + =  behavior occurs but detailed information was not collected.

† For full explanation of behaviors see Table S1 in supporting information.

*play on the ground was observed with capuchin monkeys.

BCI =  Barro Colorado Island, Panama; CV =  Corcovado National Park, Costa Rica; SR =  Santa Rosa National Park, Costa Rica; RC =  Runaway Creek Nature Reserve, Belize; PL =  Punta Laguna Reserve, Mexico.

Three of these behaviors were variants of substrate marking, which provide delayed olfactory information to conspecifics: ‘chest rub’, ‘ano-genital rub’, and ‘rub with *Ficus* root’. One was a greeting variant, ‘kiss’. Two behaviors were variants of aggressive behavior: ‘false branch shake’ and ‘overlording’. There were two variants of rare affiliative behavior, ‘interspecies grooming’ and ‘play on the ground’, and one variant of a locomotive behavior, ‘special bridging for an infant’. There were also seven variants related to food consumption choices. The remaining six variants included four drinking techniques, ‘bipedal locomotion’ and a potential thermoregulatory behavior, ‘wind catching’. Very few traits showed a similar distribution across multiple sites; however, wind catching and the consumption of rocks, mushrooms, and *Phoradendron* leaves all reached a habitual level at Santa Rosa and were absent from the other sites.

### Tradition domains

**Figure 2:**
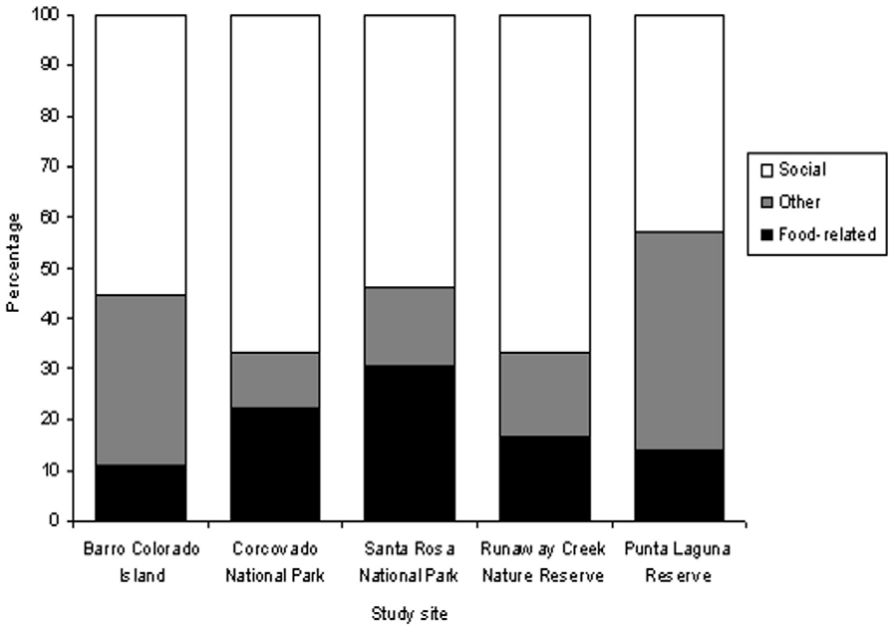
Percentage of behaviors, which met criteria for traditions, belonging to the three domain categories (social, food-related and other) at each site. See Table S1 for the division of the 22 traditions into the three domains and Figure 1 for the traditions at each site.

The occurrence of the 22 identified traditions varied across sites (Table 1). On average 57% of the identified traditions were in the social domain (Table 2). The observed bias of traditions toward the social domain is not surprising given the relative prevalence of social behaviors in the spider monkey repertoire, reflected by over half (53%) of the 62 behavior variants examined in our survey belonging to the social domain. However, this bias is still relevant from a comparative perspective when evaluating the relative occurrence of traditions in previous primate studies, where, unlike for spider monkeys, the majority belonged to the food-related domain [3], [6]. When the percentage of traditions in the social domain was calculated out of the identified number of traditions at each site, it ranged from 43% at Punta Laguna to 67% at Corcovado and Runaway Creek (Figure 2). Similar classifications across chimpanzee and orangutan (*Pongo* spp.) study sites further highlights species differences in the distribution of traditions across domains. The mean percentage of traditions in the social domain across the nine chimpanzee study sites and across the six orangutan study sites was 42% and 34% respectively, which is lower than the mean value across the five spider monkey sites (Table 2). There was, however, high variability especially across chimpanzee sites with the percentage of traditions in the social domain ranging from 0% to 64%.

**Table 2:** Mean (±SE) percentages of traditions across field sites in different domains for three primate species identified through cross-site surveys.

	Chimpanzee^[3]^	Orangutan^[6]^	Spider monkey*
**Food-related domain**	45% (±9.88)	41% (±5.66)	19% (±3.46)
**Social domain**	42% (±6.59)	34% (±9.28)	57% (±4.47)
**Other domain**	13% (±4.37)	25% (±11.50)	24% (±6.07)

*This study.

**Figure 3:**
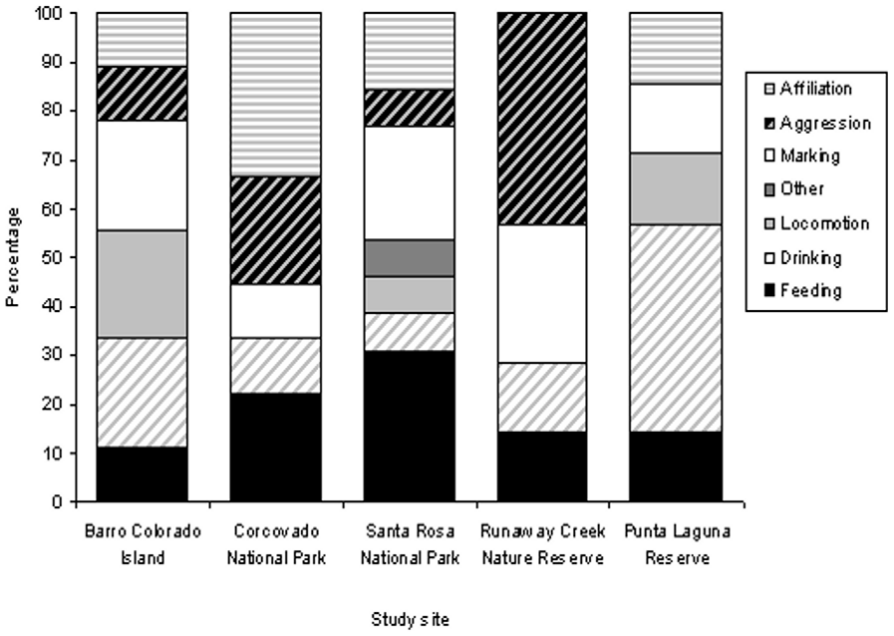
Percentage of behaviors showing evidence of tradition at each site by sub-domain category. See Table S1 for the division of behaviors into sub-domains and Figure 1 for the traditions at each site. Note that the only behavior in the sub-domain ‘Other’ was wind catching at the Santa Rosa site.

No traditions relating to aggressive interactions were recorded at the Punta Laguna site, while no traditions relating to affiliative behaviors were found at the Runaway Creek site (Figure 3). Behaviors relating to locomotion did not meet the criteria for tradition at the Corcovado and Runaway Creek sites. Candidate behaviors for traditions related to feeding, drinking and substrate marking were present across all five sites.

### Geographic distribution

**Figure 4:**
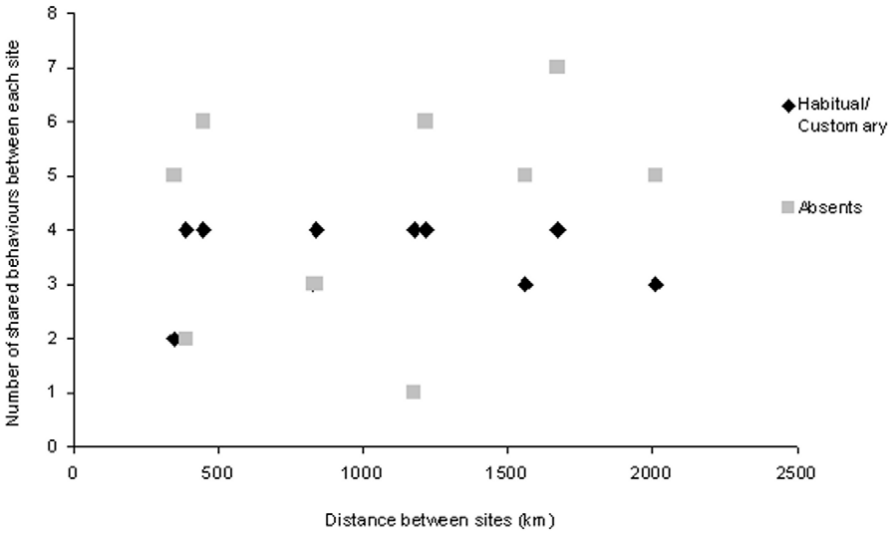
Association between the number of customary/habitual behaviors or absence of behavior each pair of study sites shared and the distance between each pair of sites.

Geographic distances between study sites range from 350 km between Runaway Creek and Punta Laguna to 2,010 km between Barro Colorado Island and Punta Laguna (Table S3). There was no significant correlation between the distance and the number of habitual or customary behaviors [*r* (10) = 0.04, *p* = 0.914], or the number of absent behaviors shared between each pair of sites [*r* (10) = 0.311, *p* = 0.282] (Figure 4).

## Discussion

We documented behavioral variation across five populations of spider monkeys providing evidence for traditions, which is relevant for a better understanding of human cultural evolution [53]. Our findings place *A. geoffroyi* alongside other species for which multiple traditions have been documented, such as humans, chimpanzees, orangutans, capuchin monkeys, bottlenose dolphins (*Tursiops* spp.), killer whales (*Orcinus orca*) and guppies (*Poecilia reticulata)* [3]–[6], [33], [35], [54]. In addition, the findings supported our two predictions. The identified traditions were more prevalent in the social domain than in other domains and included key behaviors for community identity.

### Characteristics of spider monkey traditions

A larger proportion of the 22 identified traditions belonged to the social domain. This may partially be a reflection of the species' behavioral repertoire, which includes a large number of social behaviors [39] and relatively few food processing behaviors due to their limited dexterous manipulation [42]. The overall bias of spider monkey behavior toward the social domain is also confirmed by the independent observations of each research team at the five field sites, which contributed to the selection of the 62 behaviors as appropriate candidates for traditions, over half of which belonged to the social domain.

Watson and Caldwell [55] pointed out that a large number of tradition studies have focused on food-related behaviors, either food-consumption processes during cross-site comparisons or food-rewarded behaviors in experimental procedures. This is in contrast with the bias of traditions toward the social domain in spider monkeys, which does not match the pattern reported in similar studies on orangutans and chimpanzees in which the majority belonged to the food-related domain. The 22 traditions documented here are similar in number to the 24 documented across six orangutan sites [6], but considerably fewer than the 39 documented across nine chimpanzee sites [3], [32]. It is interesting to note that the number of traditions documented per number of examined sites was relatively consistent across the three studies: 4.3 (39/9) for chimpanzees, 4 (24/6) for orangutans, and 4.4 (22/5) for spider monkeys. Thus, the inclusion of additional sites in a study seems to yield further cases of traditions, reflecting the unique repertoire of each community. A direct comparison with capuchin monkey cross-site data was not possible as separate studies focused on foraging and a sub-set of social interactions [5], [33] but it is probable that a pattern similar to that of the two great apes may apply to capuchin monkeys given that 20 variants in food processing techniques were reported across three sites [33]. Another contrast with these studies is that none of the seven food-related traditions of spider monkeys were food processing techniques.

Social behaviors have, by their very nature, a shared and often public quality, which is not necessarily the case for food-related or other subsistence-based behaviors as they are not reliant on the presence of at least one other individual to be performed [5]. This characteristic may facilitate social learning and, as a consequence, the development of traditions in the social domain. In addition, the social demands faced by species with a high degree of fission-fusion dynamics may place a greater emphasis on the functional importance of social behavior variants that are linked to group or community identity [23], [56], [57]. Support for this prediction was found as the greeting variant ‘kiss’ was identified among the traditions in our study. Additionally, variants of olfactory communication in the form of three marking behaviors were identified as traditions, further illustrating how behaviors incorporating signals of community identity might be used to convey information between frequently dispersed individuals (i.e., when in different subgroups). In some cases, such as ‘rub with *Ficus* root’ performed by multiple individuals only in the Santa Rosa community, the selection for community identity through this marking variant may have led to a form of ritual, where most subgroup members are simultaneously involved. There was variation in the percentage of traditions in the social domain (43–67%) across the five study sites. Higher variation (0–64%) occurred across chimpanzee communities. It would be interesting to assess whether such variation is associated with variation in the degree of fission-fusion dynamics across populations. Additionally, a within-population examination of behavioral variation, where genetic and ecological differences between communities are likely to be negligible, would provide more detailed evidence for such social learning opportunities and potential traditions (Santorelli et al. *in prep*.).

The community identity hypothesis to partially explain the bias of traditions toward the social domain in spider monkeys does not however account for the large number of greeting behaviors that did not meet the criteria for traditions and were listed as ‘universal’. It is noteworthy that several of these greeting variants may involve high risk, such as embraces and pectoral sniffs, which involve close body-contact and leave the recipient vulnerable to bites [27]. It is possible that the performance of these behaviors partly functions to test and strengthen relationships between community members [5]. This is in contrast to the relatively low risk ‘kiss’, reported to have reached habitual or customary status at three of the study sites, which involves less intimate contact with another individual. It is therefore possible that any variation in a high-risk greeting behavior could cause confusion, with potentially injurious consequences, especially when immigrating individuals are in the process of integrating into a new community. Innovation and dissemination of variants of low-risk social behaviors may be less problematic than innovation and dissemination of high-risk social behaviors. Thus, it might be expected that for species with a high degree of fission-fusion dynamics, for whom rapid community identity is particularly valuable, the emergence of traditions within their repertoire depends on this risk-based distinction.

### Geographic distribution pattern

Genetic variation across the site populations invariably exists. Whilst it is impossible to eliminate genetic variation in wild populations, it was minimized in this study by only examining individuals of one of the four *Ateles* species, *A. geoffroyi* [58] (see Methods). Across large geographic spaces, it is likely that inter-community genetic variation would be greatest between communities that were more geographically distant from one another. This might lead to the expectation that if genetic differences alone are responsible for explaining behavioral variation across sites, patterns of shared traditions would diminish the further apart the communities were, yet we found that geographic distance did not correlate with number of shared traditions. The failure to find a correlation, while providing no evidence of a link between genetic variation and behavioral variation, does not rule out behavioral variation due to innovation and transmission by social learning. Given that there are features of transmission processes that might affect the dispersal of socially learned behaviors between populations (i.e. immigrants as poor demonstrators of a behavior, or the transmission of a behavior performed by peers), which do not affect behaviors based on a proximate genetic cause.

The geographic distribution of traditions may reflect patterns of innovation, diffusion and transmission, which can be affected by factors such as the dispersal of individuals between communities and restrictions imposed by geographical features [6]. A loss of knowledgeable individuals, through habitat loss or hunting pressures may also affect the distribution of reported absent behaviors at a particular site over time [59]. Accordingly, a positive correlation between geographic distance and cultural difference (i.e., the percentage of shared customary and habitual variants) was found across six orangutan field sites [6]. We found no such correlation across our five field sites. Van Schaik et al. [6] suggested that the possible cause of such a correlation was a result of emigrating orangutans spreading new variants easily as they move from a site of origin to new localities. In contrast, the lack of a correlation in spider monkeys suggests strong conformity of behaviors within populations and a low likelihood of immigrants spreading new variants, as was also suggested for chimpanzee traditions [32]. Patterns of similar behavior variants are likely to emerge if behavior variants either, originate independently at multiple sites, or are introduced via immigration and then consequently spread when the costs of acquiring a particular new behavior are low [32]. However, migration does not always result in the transmission of socially learned information. In some species, such as vervet monkeys (*Chlorocebus aethiops*), individuals of the philopatric sex are preferred demonstrators of behavior than individuals of the migrating sex, and as a result of this selection highly localized traditions can emerge [60]. The social model hypothesis, which predicts primates living in structured social groups are most likely to pick knowledgeable, older or high ranking group members as demonstrators of a behavior [61], might explain such motivation. If this is the case for spider monkeys, emigrating females are unlikely to be chosen as behavior modelers and would be unlikely to transmit novel behavior variants. Although spider monkeys do not live in rigidly hierarchical social groups but in more socially tolerant communities [19], the pattern of traditions reported here similarly suggests that conformity for community specific behaviors maintain variants and, over large distances migrating females may be poor dispersers of behavior variants. The average migration distance for an emigrating female spider monkey remains unknown, but is thought to be a considerable distance (i.e., greater than four neighboring communities away [62]). Not knowing how many communities an emigrating individual passes by before settling makes it difficult to predict how closely patterns of geographic variation in traditions might reflect patterns of dispersing individuals. Consequently, limited dispersion of behavior variants between sites is more likely to result in the maintenance of site-specific behavior patterns. Finally, the lack of continuous forest across Central America limits opportunities for wide-scale dispersion of individuals and, therefore, behavior variants between communities [63], [64].

Whiten [65] suggested that cases where behavioral variants identified in one or more sites are common, but absent in at least one other site, imply that animals at the latter site are at a disadvantage. Potential self-medicating traditions may offer opportunities for case studies. For example, further cross-site research can help identify whether *Phoradendron* leaf or rock feeding conveys a selective advantage for spider monkeys in communities where it is practiced over individuals in communities where it is not practiced. The consumption of the widely available *Enterolobium cyclocarpum* fruit by individuals only in Corcovado and Punta Laguna suggests another example of a feeding variant which conveys nutritional advantages for individuals that feed upon it over individuals that do not, although, it is always possible that other communities compensate by eating another food resource [66].

Site specific consumption of widely available food resources illustrates how ecological conditions may impact to promote or hinder innovation, or the subsequent maintenance of socially transmitted behaviors. Opportunities to innovate may be influenced by the accessibility of associated substrates or social resources which may be seasonal, rare or highly unpredictable in their availability [67], [68]. For example, the fruiting cycle of *Enterolobium cyclocarpum* can be unpredictable [69]. During years when this tree does not fruit, individuals have no opportunity to innovate food processing techniques, or socially learn how to consume it. Similarly, the adaptive value of a variant or risks associated with exploration may affect innovation or social learning opportunities for some individuals more than others [68]. Therefore, despite occurrences in which social learning may account for behavioral variation across communities, the subtle interactions of ecology and personal genetic predisposition may still affect individuals' likelihood for innovation and transmission processes, contributing in part, to the establishment of traditions [67].

In other species, captive and field based experiments have been instrumental in complementing findings from cross-site comparison studies and are invaluable for exploring social learning mechanisms and transmission processes [15], [70]–[77]. The use of spider monkeys as a focal species for similar experiments would help provide evidence for the social learning mechanisms commonly used by this species and may explain the differential development of traditions across sites.

## Materials and Methods

### Ethics statement

The study was carried out in the field with free-ranging monkeys and was completely observational. Research was conducted at all times in accordance with the laws of participating countries. Approval and permission to conduct research was granted by the University of California IACUC committee, the Animal Studies Committee of Washington University # 20020071, the University of Chester Psychology Department Ethics Committee and approved by the University of Chester Animal Ethics Committee, the Animal Care Certification in compliance with the Canadian Council on Animal Care, the Costa Rica Ministry of Environment and Energy (MINAE) permit #s 418-2001-OFAU, 226-2002-OFAU and ACG-PL-030-2006, the Belize Forest Department permit # CD/60/3/09(05) and the Mexican government under the auspices of Pronatura, Peninsula de Yucatan, A.C. (PPY) # 1577105.

### Study site selection

Only field sites where research on spider monkeys of the species *A. geoffroyi* was carried out were considered in our study, in order to minimize genetic influences on any behavioral variations observed. Five sites were selected for the study (Figure S2 and Table S4), which met the following two criteria: 1) behavioral data were collected for a minimum of 12 months, in order to have a reasonable amount of observation time to document behavioral variations; and 2) the monkeys were individually recognized, so that assessment of whether individuals engaged in behavioral variants multiple times could be made, allowing for the categorization of each behavior into categories based on its prevalence at each site.

The five sites included in the survey were Barro Colorado Island, Panama (hereafter Barro Colorado [78], [79]); Corcovado National Park, Costa Rica (hereafter Corcovado [80]); Santa Rosa National Park, Costa Rica (hereafter Santa Rosa [66], [81], [82]); Runaway Creek Nature Reserve, Belize (hereafter Runaway Creek; Pavelka & Notman, unpublished data); and Otoch Ma'ax Yetel Kooh Reserve, Mexico, also known as Punta Laguna Reserve (hereafter Punta Laguna [83], [84]).

Data on two monkey communities were available at each of three sites: Corcovado, Runaway Creek and Punta Laguna. Since the aim of the survey was to examine behavioral variation across a large geographical area, responses from the two communities at each of these sites were merged.

### Survey procedure

An initial list of candidate behaviors was collated from a pilot study carried out over a two year period on the spider monkeys at the Santa Rosa and Punta Laguna field sites, as well as behaviors reported from the literature on various *Ateles* species (31,39,45–49). Then, the list was reviewed by researchers at all participating field sites and care was taken to ensure researchers accurately identified behaviors across sites. This was achieved using detailed descriptions, photographs and video clips to clarify behaviors nuances. Based on the joint feedback, additions or consolidations of behaviors were made, leading to a final list of 62 behaviors (Table S1). Survey data were compiled from data originally collected for the purpose of various behavioral studies by retrieving them from systematic records. In addition, all researchers used detailed field notes to identify patterns of rare behaviors.

The survey consisted of two phases. Phase I required researchers to document the presence or absence of each of the 62 behaviors at their field site. Categories based on the following definitions were used: *present* – behavior has occurred at the site; *absent* – behavior has never been observed at the site; *ecological explanation* - behavior has never been observed at the site but its absence is explicable by site ecology (e.g., if a particular substrate was not present at the field site, thereby removing the opportunity for behaviors associated with that substrate to occur); and *unknown* - insufficient opportunity to observe a behavior to reliably know if it was present or absent. This last category was especially relevant for behaviors that require rare conditions or might be less likely to occur in the presence of observers, despite habituation.

Phase II required researchers to classify each observed behavior at their field site using one of the categories based on the following definitions derived from Whiten et al. [3], depending on how often and by whom the behavior was performed: *customary* - behavior occurs in all or most able-bodied members of at least one age-sex class (e.g. all adult males); *habitual* - behavior is not customary but has occurred repeatedly in several individuals, consistent with some degree of social transmission; and *present* - behavior is neither customary nor habitual, but is performed multiple times by at least two individuals. Thus, in phase II performance of a behavior by only one individual at one site was classified as ‘absent’. A behavior variant was considered to be a tradition when it occurred at a habitual or customary level in at least one site while being absent in at least one other site without an ecological explanation [3], [32]. For the three sites where data from two communities were collected, the more prevalent occurrence of a behavior in each community was used for the overall site record. For example, if a behavior was ‘present’ within one community and ‘habitual’ within the other community at the same field site, the behavior was recorded as ‘habitual’ for that field site.

Pearson correlations between the number of shared customary/habitual or absent behaviors and the distance between each pair of sites were run using SPSS v.15.0.

## Supporting Information

Figure S1 ***Ateles geoffroyi*** **hand showing dramatic reduction in pollex (external thumb) (Photograph by Claire J. Santorelli).** Photograph illustrates area of reduced pollex on the left hand.
(TIF)

Figure S2 **Map of Central America showing locations of the five field sites participating in the study.** Arrows illustrate location of participating field sites within their host country.
(TIF)

Table S1 **Definitions and domains of the 62 behaviors considered in the study.**
(DOC)

Table S2 **Prevalence of behavior variants across study sites.**
(DOC)

Table S3 **Distance (kilometers) between sites (using Google Earth ruler,** http://earth.google.com **).**
(DOC)

Table S4 **Site information.**
(DOC)
