# Supplementary figures and images for: Traditions in Spider Monkeys Are Biased towards the Social Domain
Source: PLoS One. 2011 Feb 23;6(2):e16863. doi: 10.1371/journal.pone.0016863 (PMC3044143; doi:10.1371/journal.pone.0016863)

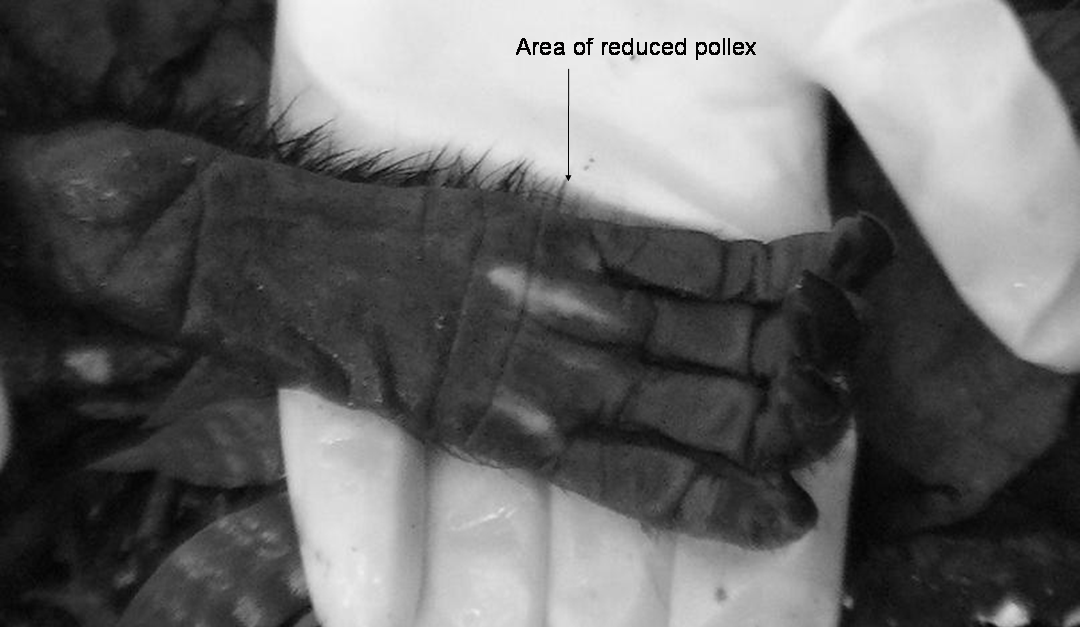

Supplement: Figure S1 — Ateles geoffroyi hand showing dramatic reduction in pollex (external thumb) (Photograph by Claire J. Santorelli). Photograph illustrates area of reduced pollex on the left hand. (TIF) [file pone.0016863.s001.tif]

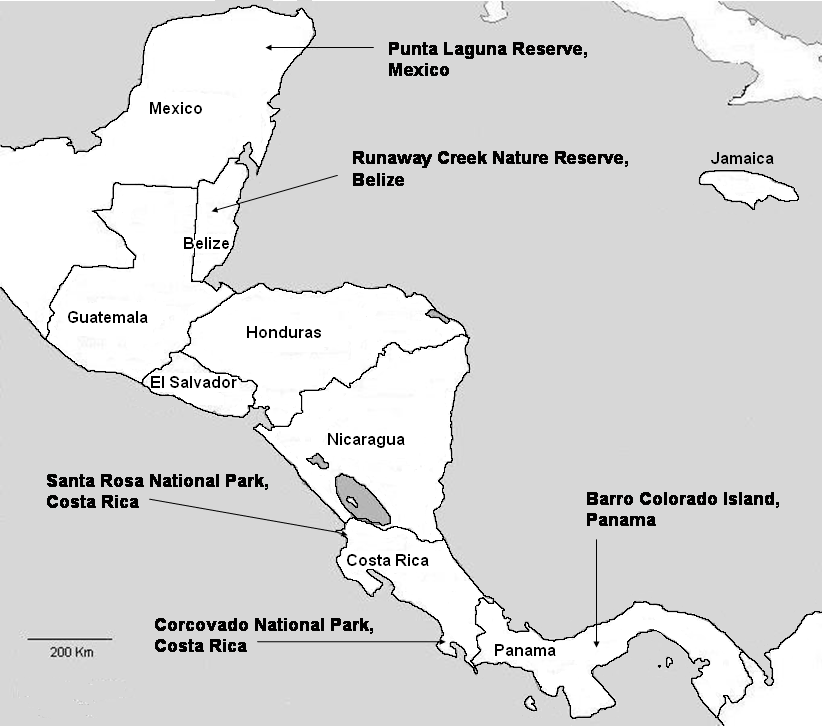

Supplement: Figure S2 — Map of Central America showing locations of the five field sites participating in the study. Arrows illustrate location of participating field sites within their host country. (TIF) [file pone.0016863.s002.tif]
