# Supplementary material for: Traditions in Spider Monkeys Are Biased towards the Social Domain
Source: PLoS One. 2011 Feb 23;6(2):e16863. doi: 10.1371/journal.pone.0016863 (PMC3044143; doi:10.1371/journal.pone.0016863)
Supplement: Table S1 — Definitions and domains of the 62 behaviors considered in the study. (DOC) [file pone.0016863.s003.doc]

Table S1. Definitions and domains of the 62 behaviors considered in the study.

| **Behavior** | **Definition** | **Domain^Ŧ^** | **Sub-domain^±^** |
| --- | --- | --- | --- |
| Consume *Enterolobium cyclocarpum* fruit^†^ | Individual consumes *Enterolobium cyclocarpum* fruit. | Food-related | Feeding |
| Consume *Phoradendron* leaves^†^ | Individual consumes *Phoradendron* leaves, commonly known as mistletoe leaves. | Food-related | Feeding |
| Consume *Phoradendron* fruit^†^ | Individual consumes *Phoradendron* fruit, commonly known as mistletoe berries. | Food-related | Feeding |
| Consume *Anacardium excelsum* pith^†^ | Individual consumes *Anacardium excelsum* pith. | Food-related | Feeding |
| Consume *Orbignya cohune* | Individual consumes *Orbignya cohune*, commonly known as cohune palm nuts. | Food-related | Feeding |
| Consume *Stemmadenia donnellsmithii* | Individual consumes *Stemmadenia donnellsmithii*, commonly known as horseball fruit. | Food-related | Feeding |
| Consume *Metopium browneii* | Individual consumes *Metopium browneii*, commonly known as black poisonwood fruit. | Food-related | Feeding |
| Consume *Acacia* (spp.) | Individual consumes *Acacia* fruit, commonly known as bullhorn acacia legumes. | Food-related | Feeding |
| Consume cuckoo spit (*Philaenus* spp.) | Individual consumes cuckoo spit which is a foam with often developing nymphs inside. | Food-related | Feeding |
| Consume mushrooms^†^ | Individual consumes epiphytic mushrooms or ground mushrooms. | Food-related | Feeding |
| Consume bark | Individual consumes tree bark. | Food-related | Feeding |
| Consume caterpillars/larvae^†^ | Individual consumes caterpillars or larvae. | Food-related | Feeding |
| Consume bees | Individual consumes bees either directly or on leaves which are also consumed. | Food-related | Feeding |
| Consume other insect | Individual consumes insects other than bees. | Food-related | Feeding |
| Consume rocks^†^ | Individual consumes rocks, collected from the ground or from accessible cliff faces. | Food-related | Feeding |
| Consume soil | Individual consumes soil. | Food-related | Feeding |
| Lick water off fist | Individual drinks water by putting their hand in water source and then licks it off their closed fist. | Other | Drinking |
| Lick water off palm^†^ | Individual drinks water by putting their hand in water source and then licks it off their open palm. | Other | Drinking |
| Lick water from leaf sponge | Individual drinks water by putting their hand in water source and then pulls out a handful of leaves from the source and sucks/licks the water from the leaves. | Other | Drinking |
| Cup water in hand | Individual drinks water by putting their hand in water source and then sucks/licks it from a cupped hand. | Other | Drinking |
| Dribble water into mouth^†^ | Individual drinks water by putting their hand/tail/leaf into water source and then dribbles the water directly into their mouth. | Other | Drinking |
| Drink using head only^†^ | Individual sticks it's head directly into water source to suck or lap up the water directly. | Other | Drinking |
| Drink from ground waterhole/lake^†^ | Individual obtains water from a large ground source such as a lake or large pond. | Other | Drinking |
| Regular bridging for an infant | Mother (or another individual) uses their body to 'bridge' between two trees in the canopy in order for an infant to climb over their body to cross the gap. | Social | Locomotion |
| Special bridging for an infant^†^ | Aim is as above, but the mother bridges in a different way, either by passing over the area first and then returning to bridge when the infant does not follow her easily; or the mother uses her hands to reach out to the infant and help them cross a gap; or the mother uses her weight to tip the canopy branch to where the infant is waiting, and the infant crosses without coming into direct contact with the mothers body. | Social | Locomotion |
| Vaulting | Individual uses their momentum to rock backwards and forwards on a flexible tree (small tree and not necessarily the tip) to move it closer to an adjacent tree for them to jump/pull themselves onto. | Other | Locomotion |
| Travel on ground | Individual comes down to the ground to cross an area where there is no canopy cover, or Individual moves on the ground to escape another individual. | Other | Locomotion |
| Play on ground^†^ | During intra-group play, individual comes down to the ground and uses it as a play substrate. | Social | Affiliation |
| Bipedal walking^†^ | Individual walks upright, either on the ground or on a branch whilst not holding onto any other substrate. | Other | Locomotion |
| Rub with *Ficus* root^†^ | Individual rubs the tips of *Ficus* aerial roots predominantly on their upper chest region, or under the arms, but may also be on other regions. This may follow biting or eating of the aerial roots, and be followed by chest rubbing on a nearby branch substrate. | Social | Marking |
| Leaf rubbing | Individual chews leaves and then rubs the leaves (and/or saliva) predominantly on their upper chest region, or under the arms, but may also be on other regions. This may be followed by chest rubbing on a nearby branch substrate. | Social | Marking |
| Chest rub^†^ | Individual rubs their upper chest region on a branch. This may follow sniffing of the branch first. | Social | Marking |
| Ano-genital rub^†^ | Individual rubs their ano-genital region on a branch. This may follow sniffing of the branch first. | Social | Marking |
| Overmark | Individual chest rubs or ano-genital rubs on the exact spot that another monkey either chest rubbed, ano-genital rubbed or sat on. | Social | Marking |
| Wind catching^†^ | Individual sits in top of tree and orientates head in direction of a gust of wind with mouth wide open. | Other | Other |
| Cave dwelling | Individuals enter inside a cave. | Other | Other |
| Solicit for groom | Individual presents a specific body part to or lies close, draping themselves across another individual for grooming. | Social | Affiliation |
| Leg raised grooming (self grooming | Individual lifts their leg directly to the side of their head, keeping it straight, while manipulating the fur with its own hands or mouth, instead of having leg bent and by the side of their head. | Other | Other |
| Leg raised grooming (allo-grooming) | Individual lifts their leg directly to the side of another individual’s head, keeping it straight, and the other individual manipulates the fur of the other with its hands or mouth, instead of having it bent and by the side of the groomees head. | Social | Affiliation |
| Wound cleaning | Individual touches or licks the wound of another individual. | Social | Affiliation |
| Interspecies grooming^†^ | Individual manipulates fur of another species with its own hands or mouth or has their own fur manipulated by the hand or mouth of another species. | Social | Affiliation |
| Allo-carrying | Individual carries an infant or juvenile who is not their offspring. | Social | Affiliation |
| Allo-nursing | Individual nurses an infant or juvenile who is not their offspring. | Social | Affiliation |
| Embrace using one arm | Individual wraps one arm around the shoulder or back of another individual. | Social | Affiliation |
| Embrace using two arms | Individual wraps two arms around the shoulders or back of another individual. | Social | Affiliation |
| Pectoral sniff of another | Individual places its nose at the chest or arm pit region of another. This might follow having their head guided into the chest or arm pit region by the other individual. | Social | Affiliation |
| Kiss^†^ | Face to face behaviour in which two individuals get their faces close cheek-to-cheek (as in a human kiss on the cheek), usually with no contact or only minor contact of the cheeks between the two individuals. | Social | Affiliation |
| Face greet of another | Individual gazes in the direction of another individual and purses lips outward in a wide kiss-like gesture. | Social | Affiliation |
| Face touch of another | Individual places their hand, hands or fingers gently on the face of another individual. | Social | Affiliation |
| Grappling | Sequence of intense embraces, tail wrapping, face touches between two individuals, often accompanied by loud vocalizations and approaching and retreating. | Social | Affiliation |
| Intertwined tails | Two individuals sit close to each other with intertwined tails like titi monkeys. | Social | Affiliation |
| Arm wrapping threat | One individual wraps its arm around another at shoulder level while threatening another monkey, animal, observer or nothing obvious. | Social | Aggression |
| Mutual arm wrapping threat | Similar to the above category but the other individual also wraps their arm around the first individual in a mutual arm wrap. Individuals may growl and/or shake branches as well. | Social | Aggression |
| Branch shake | Individual vigorously moves a branch up and down at another monkey, animal, observer or nothing obvious. | Social | Aggression |
| Mutual branch shake | Two or more individuals vigorously move branches up and down at another monkey, animal, observer or nothing obvious, whilst in contact or proximity with one another. | Social | Aggression |
| False branch shake^†^ | Individual sways its chest and shoulders back and forth in a manner similar to movements with branch shaking but no branches are shaken. | Social | Aggression |
| Branch break | One or more individuals snaps off tree branches and drops or throws them at another monkey, animal, observer or nothing obvious. | Social | Aggression |
| Raiding | As described by Aureli et al. (2006). Individuals (so far only reported in males) descend to the ground and move in single file, silently into the territory of neighboring communities. | Social | Aggression |
| Overlording^†^ | 2+ individuals pile on top of one another in a threat display aimed towards an observer or another monkey. One individual climbs on the back of another and wraps legs and arms around the individual’s torso. | Social | Aggression |
| Interspecies play | Individual engages in affiliative wrestling, slapping or mock biting with another species. | Social | Aggression |
| Interspecies aggression | Individual is involved with a hostile, non-affiliative encounter with another species which might include chasing, threatening or physical aggression. | Social | Aggression |
| Interspecies allo-care | Individual is involved with carrying an infant of another species. | Social | Aggression |

^Ŧ^Social domain – behavior involved a direct or indirect interaction with another individual, or functioned as a form of vocal or olfactory communication; Food-related domain – behavior involved handling or ingestion of a substrate; Other domain – behavior could not be categorized within either the social or food-related domains.

±Feeding sub-domain – behavior involved handling or ingestion of a substrate; Affiliation sub-domain – behavior involved an affiliation interaction with another individual; Aggression sub-domain – behavior involved an aggression interaction with another individual; Marking sub-domain – behavior involved rubbing a substrate using the chest, mouth or ano-genital region, or rubbing the body with a plant substrate which may or may not be followed by another marking behavior; Locomotion sub-domain – behavior focused on using a specific mechanism of movement to cover an area; Other sub-domain – behavior could not be categorized within any other sub-domain category.

^†^Behavior identified as a tradition (see text for criteria).
