# Supplementary material for: Traditions in Spider Monkeys Are Biased towards the Social Domain
Source: PLoS One. 2011 Feb 23;6(2):e16863. doi: 10.1371/journal.pone.0016863 (PMC3044143; doi:10.1371/journal.pone.0016863)
Supplement: Table S2 — Prevalence of behavior variants across study sites. (DOC) [file pone.0016863.s004.doc]

**Table S2. Prevalence of behavior variants across study sites.**

| **Band** |  | **Behavior^†^** | **Prevalence** | | | | |
| --- | --- | --- | --- | --- | --- | --- | --- |
|  |  |  | **BCI** | **CV** | **SR** | **RC** | **PL** |
| A | 1 | Intertwined tails | A | A | A | A | A |
|  | 2 | Allo-nursing | A | A | A | A | A |
|  | 3 | Interspecies allo-care | A | A | A | A | A |
|  | 4 | Leg raised grooming (allo-grooming) | A | A | A | A | A |
|  | 5 | Leg raised grooming (self-grooming) | A | A | A | A | A |
|  | 6 | Lick water from leaf sponge | A | A | A | A | A |
|  | 7 | Cup water in hand | A | A | A | A | A |
|  | 8 | Consume bees | A | A | A | A | A |
|  | 9 | Consume bark | A | A | A | A | A |
|  | 10 | Consume other insect | A | A | A | A | A |
| B | 11 | Face touch of another | A | P | P | A | A |
|  | 12 | Overmark | A | A | P | P | A |
|  | 13 | Cave dwelling | E | E | A | P | A |
|  | 14 | Consume *Stemmadenia donnellsmithii* | E | A | A | P | E |
|  | 15 | Consume *Acacia* (spp.) | E | P | A | P | A |
|  | 16 | Consume soil | + | A | A | P | A |
| C | 17 | Raiding | E | C? | U | U | C |
|  | 18 | Consume *Orbignya cohune* | E | E | E | H | E? |
|  | 19 | Consume *Metopium browneii* | E | E | E | C | C |
|  | 20 | Consume cuckoo spit (*Philaenus* spp.) | U | U | H | U | P |
| D | 21 | Pectoral sniff of another | H | C | C | H | H |
|  | 22 | Wound cleaning | P | H | H | H | P |
|  | 23 | Interspecies play | P | C | H | P | P |
|  | 24 | Embrace using one arm | + | + | C | H | C |
|  | 25 | Embrace using two arms | + | + | C | H | P |
|  | 26 | Face greet of another | + | + | H | P | H |
|  | 27 | Grappling | H | H | H | H | P |
|  | 28 | Solicit for groom | + | + | H | H | C |
|  | 29 | Allo-carrying | P | H | H | P | P |
|  | 30 | Regular bridge for an infant | + | + | P | H | H |
|  | 31 | Interspecies aggression | H | C | C | P | P |
|  | 32 | Branch shake | H | C | H | H | H |
|  | 33 | Branch break | H | P | H | H | P |
|  | 34 | Mutual arm wrapping threat | H | H | H | H | H |
|  | 35 | Arm wrapping threat | H | H | H | H | P |
|  | 36 | Mutual branch shake | H | H | H | H | H |
|  | 37 | Leaf rubbing | H | H | C | P | P |
|  | 38 | Lick water off fist | H | + | C | C | C |
|  | 39 | Vaulting | H | C | P | H | H |
|  | 40 | Travel on ground | H | H | P | H | C |

Band A = behaviors absent across all sites; Band B = behaviors absent from the majority of sites, or present at one or two but not to the extent of being habitual or customary; Band C = behaviors were customary or habitual at some sites, or a behaviors’ absence was explicable due to an ecological explanation, or it was unknown if required substrate was available at a site; Band D = behaviors met criteria for traditions. BCI = Barro Colorado Island; CV = Corcovado; SR = Santa Rosa; RC = Runaway Creek; PL = Punta Laguna. C = customary; H = habitual; P = present; A = absent; E = ecological explanation (i.e. a required environmental component did not occur at the field site: e.g. no caves for ‘cave dwelling’; no tree species for food consumption variants; in the case of ‘raiding’ the BCI community had no neighboring community); U = unknown (see text for definition of each category); + = behaviour occurs but detailed information not collected; ? = behavior category suspected but more contextual information required to confirm. **^†^**For full explanation of behaviors see Table S1.
