# Supplementary material for: Traditions in Spider Monkeys Are Biased towards the Social Domain
Source: PLoS One. 2011 Feb 23;6(2):e16863. doi: 10.1371/journal.pone.0016863 (PMC3044143; doi:10.1371/journal.pone.0016863)
Supplement: Table S3 — Distance (kilometers) between sites (using Google Earth ruler, http://earth.google.com ). (DOC) [file pone.0016863.s005.doc]

Table S3. Distance (kilometers) between sites (using Google Earth ruler, http://earth.google.com).

|  | Barro Colorado Island | Corcovado | Santa Rosa | Runaway Creek | Punta Laguna |
| --- | --- | --- | --- | --- | --- |
| Barro Colorado Island | - | 450 | 840 | 1,670 | 2,010 |
| Corcovado |  | - | 390 | 1,215 | 1,560 |
| Santa Rosa |  |  | - | 830 | 1,175 |
| Runaway Creek |  |  |  | - | 350 |
| Punta Laguna |  |  |  |  | - |
