# Supplementary material for: Traditions in Spider Monkeys Are Biased towards the Social Domain
Source: PLoS One. 2011 Feb 23;6(2):e16863. doi: 10.1371/journal.pone.0016863 (PMC3044143; doi:10.1371/journal.pone.0016863)
Supplement: Table S4 — Site information. (DOC) [file pone.0016863.s006.doc]

Table S4. Site information

| Site location | **Barro Colorado Island, Panama** | **Corcovado National Park, Costa Rica** | **Santa Rosa National Park, Costa Rica** | **Runaway Creek Nature Reserve, Belize** | **Punta Laguna (Otoch Ma'ax yetel Kooh) Reserve, Mexico** |
| --- | --- | --- | --- | --- | --- |
| Community size^Ŧ^ | 21 | ≤101 | 26-32 | 1) 30-34; 2) 15-20 | 1) 22-24; 2) 30-39 |
| Community composition^‡^ | 4-5 adult males  7 adult females  0 subadult males  3 subadult females  2 juvenile males  3 juvenile females  0 infant males  2 infant females  1 infant unknown sex | Community 1:  20 adult males  36 adult females  8 subadult males  10 subadult females  8 juvenile males  5 juvenile females  8 infant males  6 infant females  Community 2:  17 adult males  13 adult females  2 subadult males  4 subadult females  3 juvenile males  2 juvenile females  2 juveniles unknown sex  1 infant male  1 infant female  4 infants unknown sex | 1-8 adult males  8-9 adult females  2 sub adult males  6-8 sub adult females  1 juvenile male  1-4 juvenile females  3-5 infant males  0-2 infant females | Community 1:  5 adult males  13 adult females  1 subadult male  3 subadult females  5 juvenile males  5-6 juvenile females  1 infant male  3 infant females  Community 2:  8 adult males  9 adult females  1 subadult male  5 subadult females  3 juvenile males  3 juvenile females  2 infants unknown sex | Community 1:  1-4 adult males  8 adult females  1-3 subadult males  2-3 subadult female  2-3 juvenile males  2 juvenile females  3-5 infant males  1-2 infant females  Community 2:  4-5 adult males  8-14 adult females  1-2 subadult male  1-4 subadult females  2-3 juvenile male  3-6 juvenile females  2 infant males  1-4 infant females  2 infants unknown sex |
| Observation period | October 1997-December 1998 | November 2002-October 2003 | September 2003-May 2007 | January 2008 – April 2009 | December 2003-April 2004, October-December 2004; January-May 2006; May-September 2007 |
| Annual rainfall | 2600mm | 5657mm | 1,600mm | unknown | 1,500mm |
| Wet season | May - December | April - November | May - December | June - December | May - November |
| Dry season | January - March | December - March | January - April | January - May | December - April |
| Estimated home range | 9.2 km² | 5 km² | 3.5 km^2^ | Community 1: 1.34 km² Community 2: 2.12 km² | Community 1: 0.95km²  Community 2: 1.66km² |
| Minimum hours of observation^±^ | 1200 | 1146 | 1219 | 1000 | 1295 |

^Ŧ^Range of individuals refers to minimum and maximum community composition size over the course of data collection due to demographic changes.

^*^Data from three field sites were collected from two communities, ‘1)’ refers to the 1st community and ‘2)’ refers to 2nd community.

±Minimum hours of observation were calculated from focal animal samples; this is a very conservative measure as the actual hours of observation were considerably more because communities were followed for extensive periods without collecting focal animal samples.

**^‡^**Age classes were defined as follows: Adult, >8 yrs; Subadult, 5-8 yrs; Juvenile, 3-5 yrs; and Infant, 0-3 yrs or until their mother had another infant, if earlier. In cases when birth date was unknown, size was used to estimate age and subadults and immigrant females were classed as sexually mature individuals who moved independently from the mother and in different subgroups, but were not fully adult size.
